# Evaluation of the Validity, Reliability, and Kinematic Characteristics of Multi-Segment Foot Models in Motion Capture

**DOI:** 10.3390/s20164415

**Published:** 2020-08-07

**Authors:** Yuka Sekiguchi, Takanori Kokubun, Hiroki Hanawa, Hitomi Shono, Ayumi Tsuruta, Naohiko Kanemura

**Affiliations:** 1 Graduate Course of Health and Social Services, Graduate School of Saitama Prefectural University, Saitama 343-8540, Japan; 2191005s@spu.ac.jp; 2 Research Fellowship for Young Scientists, Japan Society for the Promotion of Science, Tokyo 102-0083, Japan; 3 Department of Health and Social Services, Saitama Prefectural University, Saitama 343-8540, Japan; kokubun-takanori@spu.ac.jp; 4 Department of Rehabilitation, Faculty of Health Science, University of Human Arts and Sciences, Saitama 339-8539, Japan; hiroki_hanawa@human.ac.jp; 5 Yatsuka Seikeigekanaika, Saitama 343-0028, Japan; 2081304e@spu.ac.jp; 6 Ageo Futatsumiya Clinic, Saitama 362-0017, Japan; 2081307f@spu.ac.jp

**Keywords:** reproducibility of results, bias, kinematics, foot, optical motion capture

## Abstract

This study aimed to evaluate the validity and reliability of our new multi-segment foot model by measuring a dummy foot, and examine the kinematic characteristics of our new multi-segment foot model by measuring the living body. Using our new model and the Rizzoli model, we conducted two experiments with a dummy foot that was moved within a range from −90 to 90 degrees in all planes; for the living body, 24 participants performed calf raises, gait, and drop jumps. Most three-dimensional (3D) rotation angles calculated according to our new models were strongly positively correlated with true values (*r* > 0.8, *p* < 0.01). Most 3D rotation angles had fixed biases; however, most of them were in the range of the limits of agreement. Temporal patterns of foot motion, such as those in the Rizzoli model, were observed in our new model during all dynamic tasks. We concluded that our new multi-segment foot model was valid for motion analysis and was useful for analyzing the foot motion using 3D motion capture during dynamic tasks.

## 1. Introduction

The human foot consists of 26 bones and 33 joints, the fine movements of which make various motion tasks efficient. A detailed grasp of the pronation and supination of the foot is important for evaluating foot function. The functions of the foot are mainly shock absorption and the exertion of power, and the two contradictory functions of the foot occur even while walking. The pronation of the foot has been observed during the initial-contact to mid-stance phases of walking [1,2]. The pronation of the talocalcaneal joint makes the axes of motion of the talonavicular and calcaneocuboid joints parallel to each other, thus increasing the flexibility of the foot [3]. The pronation of the foot has been observed in the terminal phase of walking [1,2]. The supination of the talocalcaneal joint intersects the axes of motion of the talonavicular and calcaneocuboid joints, thus increasing the stability of the foot [3]. In this context, the pronation and supination of the foot contribute to efficient movement. However, the model conventionally used in most motion analyses, e.g., the plug-in-gait model [4], treats the foot as a single segment, and ignores the internal foot motion [5]. Therefore, the inability to capture performance-related motion is a major limitation. In recent years, the multi-segment foot model was proposed. It is capable of detailed motion analysis and has helped advance foot research. Among various multi-segment foot models, the Oxford foot model [6], the Heidelberg foot measurement method [7], and the Rizzoli foot model proposed by Leardini [1] are currently being used. These multi-segment foot models demonstrated repeatability using various methods [8,9,10]. They have been applied clinically to patients with cerebral palsy [11,12], flat foot [13,14], hallux valgus [15], and diabetes-related foot deformity [16]. Additionally, the reproducibility of foot bone kinematics and the effect of skin artifact have been evaluated using bone pins [17] and fluoroscopy [18] because the internal joint of the foot motion is very small and complex. However, little has been reported regarding the validity and reliability of the multi-segment foot model.

Using the multi-segment foot model, Dustin [19] reported not only foot kinetics but also kinematics by grounding the foot on two force plates [20]. Dustin’s model is defined as a shank and three foot segments; the hallux, forefoot, and rearfoot as the sagittal planes, and it has also been evaluated for repeatability and segment rigidity [19]. Leardini [1] reported foot kinematics using the Rizzoli foot model. The Rizzoli model is defined by a shank and four foot segments: hallux, forefoot, midfoot, and rearfoot [1,2]. This model is able to capture inversion and eversion in more detail because the segments are defined as triangular surfaces in the transverse planes [3,18]. However, on examining foot kinetics using the Rizzoli model, an inter-segment moment or power can only be estimated by combining both data using a force plate and a foot pressure mat [21]. In addition, a study that examined the differences of multi-segment foot models reported that kinematic patterns depend on the location of tracking markers using the rigid-body assumption [22].

Therefore, in order to analyze the foot kinetics and kinematics in the frontal plane in detail during dynamic movements, we created a multi-segment foot model which has three foot segments as the transverse planes by referencing Dustin’s foot model [19] and the Rizzoli foot model [1,2]. Regarding the rearfoot, the segment defines two patterns, the transverse and sagittal planes. The first purpose of this study was to validate the reliability of our new multi-segment foot model by measuring a dummy foot with markers attached according to our new model. The second purpose was to examine the kinematic characteristics of our new multi-segment foot model by measuring the living body with markers attached. Our new model that allows the detailed analysis of the foot kinetics and kinematics in the frontal plane during dynamic movements might not only demonstrate foot motor function and disorder mechanism, but also might be able to contribute to the design of braces and artificial limbs that assist foot function.

## 2. Materials and Methods

**Figure 1:**
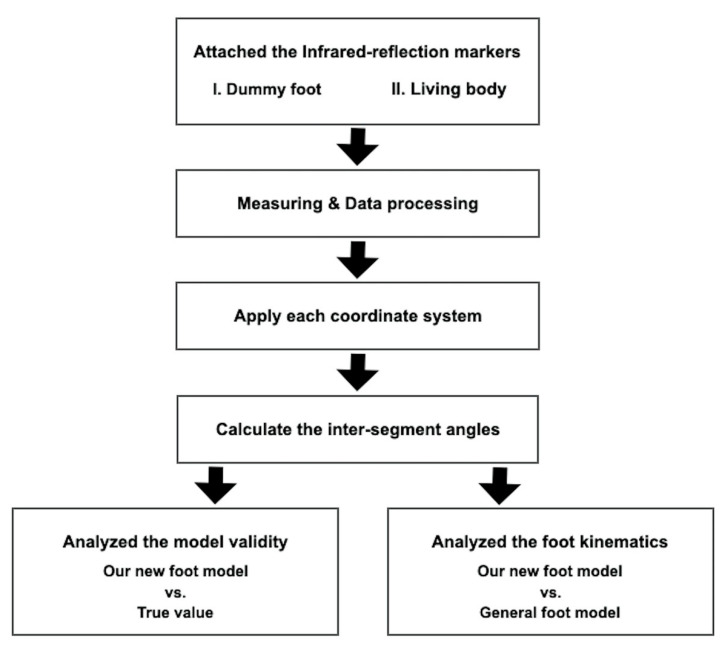
The process flow chart.

We proposed a new multi-segment foot model and conducted two experiments by measuring a dummy foot and the living body, respectively. The process flow chart is shown in Figure 1. In the following sections, the materials and methods are described in detail.

### 2.1. Multi-Segment Foot Model

**Figure 2:**
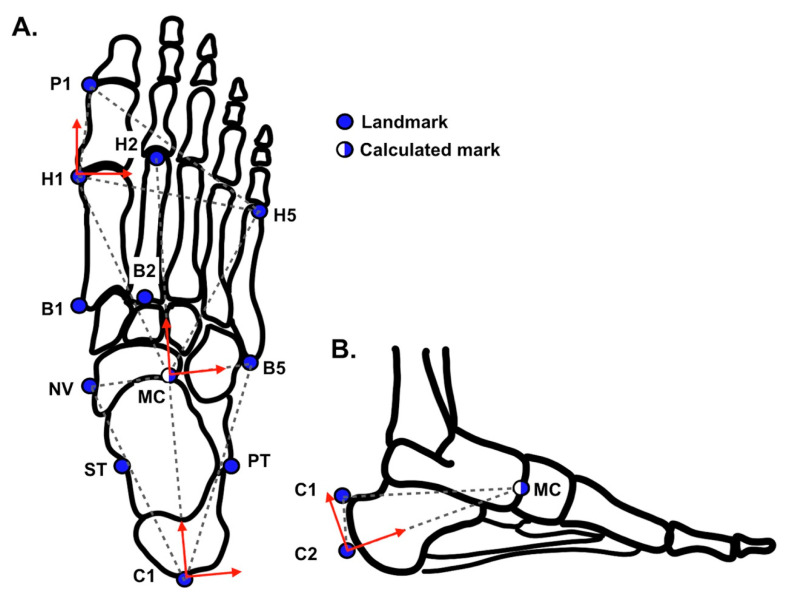
Marker location, segment reference planes (dash triangles), and the primary- and tertiary-axis (red solid arrows) on these planes are shown. Rear-foot segment references in the planes of two conditions; transverse plane (**A**) and sagittal plane (**B**).

This study used three multi-segment foot models; our new model, our new model_2, and the Rizzoli model. Our new model consists of a shank that includes the tibia and fibula and three foot segments: (1) the hallux, including distal and proximal phalanx; (2) the forefoot, including the five metatarsal bones; and (3) the rearfoot; including the navicular, lateral, middle, and medial cuneiforms, cuboid, calcaneus, and talus. Marker location and segment reference frames are shown in Table 1 and Table 2 and Figure 2A. Second, our new model_2 has the same rigid segments, marker locations, and segment reference frames as the new model, except for the reference frame of the rearfoot. The reference frame of the Rearfoot is shown in Table 2 and Figure 2B. Third, we used the Rizzoli model [1,2] as a generalized multi-segment foot model.

### 2.2. Dummy Foot

In order to calculate the true value when treating the foot as a rigid-multi-segments, and to examine the validity and reliability of measuring foot motion using our new model, we made a dummy foot of styrene foam and divided it into three segments: (1) the toe with a height of 3 cm, a width of 8 cm, and a length of 5.5 cm; (2) a forefoot with a height of 5 cm, a width of 8 cm, and a length of 9.5 cm; and (3) a rearfoot with a height of 5 cm, a width of 6.5 cm, and a length of 7.5 cm.

### 2.3. Participants

To examine the kinematic characteristics of our new foot model in vivo with markers attached according to our new model, we recruited 24 healthy adults who were not training or engaging in sports regularly. Eleven men with a mean age of 24.1 (standard deviation (SD) 2.4) years, a mean height of 172.5 (SD 3.8) cm, and a mean weight of 66.5 (SD 8.1) kg, and 13 women with a mean age of 23.1 (SD 0.8) years, a mean height of 162.5 (SD 4.1) cm, and a mean weight of 53.8 (SD 3.8) kg participated in this study. Participants were excluded if they had a history of orthopedic, neurological, and/or musculoskeletal disorders likely to affect their calf raise or drop jump. All participants provided written informed consent following a detailed explanation of the study’s purpose and risks involved according to the Declaration of Helsinki. The study was approved by the Ethics Committee on Human Experimentation at Saitama Prefectural University (Approval Number 29,508).

### 2.4. Experimental Protocol of Dummy Foot

**Figure 3:**
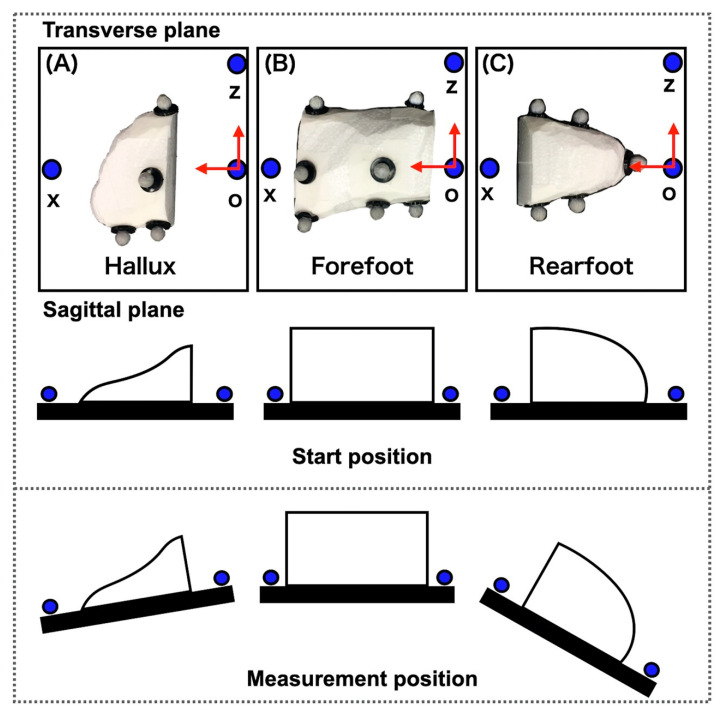
Three infrared-reflecting markers (point o, point x, and point z) were attached, respectively, to the three plates that were placed on each dummy foot to calculate the true value. The X and Z axes (red solid arrows) on these plates were shown. Angles for the plates were defined as the true values for the corresponding joint angles; the angle of plate (**A**) relative to plate (**B**) corresponded the Hallux relative to forefoot (Met_Hal) angle and the angle of plate (**B**) relative to plate (**C**) corresponded the forefoot relative to rearfoot (Cal_Met) angle. Measurement positions were from −90 to 90 degrees in all planes.

Infrared-reflecting markers (diameters 9.5 mm) were attached to 17 landmarks on the dummy foot according to our new foot model. Markers shared across two segments such as the dorso-medial aspect of the first metatarsal head, dorso-medial aspect of the second metatarsal head, dorso-lateral aspect of the fifth metatarsal head, dorso-lateral aspect of the fifth metatarsal base, and the most medial apex of the navicular bone were attached as markers to each segment in duplicates, because the dummy foot consisted of three separate segments. Each segment of the dummy foot was placed on the plate in which three infrared-reflecting markers (point o, point x, and point z) were attached, such that the coordinate system of the plate could be defined; the X-axis was from point o to point x, the Z-axis was from point o to point z, and the Y-axis was orthogonal to the previous two. Each segment at both ends (the toe and rearfoot) was measured by moving the corresponding plate with them, within a range from −90 to 90 degrees in all planes from 5 to 10 degrees (Figure 3).

### 2.5. Experimental Protocol of Living Body

**Figure 4:**
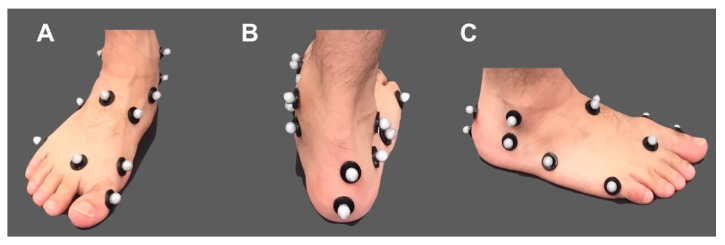
The right foot which attached the infrared-reflection markers, shown from the front (**A**), from the back (**B**), and from the outside (**C**).

Infrared-reflecting markers (diameters 14 and 9.5 mm) were attached to 65 landmarks on the participants, according to the Vicon plug-in-gait full-body and our new foot model (Figure 4). The markers were placed on the following land-marks: bilateral forehead, occipital region, acromion, lateral arm, lateral elbow joint, lateral forearm, ulnar styloid, radial styloid, second metacarpal head, superior anterior iliac crest, posterior superior iliac crest, lateral thigh, lateral knee joint, rough tibia, calcaneal head, lateral lower thigh, medial and lateral malleoli, posterior calcaneus, Achilles tendon attachment, sustentaculum tali, peroneal trochlea, navicular tuberosity, the bases of the first, second and fifth metatarsals, head of the first, second and fifth metatarsals, and the proximal phalanx of the hallux, and seventh cervical lumbar spine, sternum, xiphoid, right shoulder blade, and the tenth thoracic spine. Participants performed two dynamic tasks: (1) a calf raise performed by raising the heel as far as possible with both feet standing; (2) gait at a comfortable speed; and (3) a drop jump task that entailed a high vertical jump, immediately after landing on both feet from a jump from a 40 cm-high box. The subjects began the task with a comfortable timing and sufficiently practiced the task before measurement. Each participant performed one trial of five calf raises, three trials of a gait, and seven trials of a drop jump, with sufficient rest between the trials.

### 2.6. Data Collecting

Trajectories of surface markers were collected using the Vicon Nexus 2.2.10, a three-dimensional (3D) motion analysis system (Vicon, Oxford, UK) with 17 infrared cameras at 100 Hz. All data were synchronized using Vicon Workstation v4.5 software and saved for offline analysis. We analyzed all data using MATLAB 2018a (The MathWorks, Natick, MA, USA).

### 2.7. Data Analysis

Three-dimensional inter-segment rotation angles were calculated using motion analysis system software (Visual 3D, C-motion, Germantown, MD). A joint coordinate system of each segment was defined according to Table 2 for our new model, Figure 3 for the true value in the experiment of a dummy foot, and the literature [1,2] for the Rizzoli model in the experiment of a living body. The Euler angles were calculated by rotating in the Z-, X-, and Y- axes: dorsi-/plantar-flexion (Df/Pf) as the rotation about the Z-axis, eversion/inversion (Ev/Inv) about the X-axis, and abduction/adduction (Abd/Add) about the axis orthogonal to the other two. The angles for the plates were defined as the true values for the corresponding joint angles; the angle of plate A relative to plate B corresponded the Hallux relative to forefoot (Met_Hal) angle and the angle of plate B relative to plate C corresponded the forefoot relative to rearfoot (Cal_Met) angle (Figure 3). The joint angles were subtracted from the means of the joint angles during the static standing position for 15 s. Displacement data were filtered using a zero-phased lag, fourth-order Butterworth filter with a cutoff frequency of 6 Hz. We extracted the peak value during the calf raises and drop jumps that were the slowest and fastest movements, respectively, in the dynamic tasks. The definitions of abbreviations for angle names according to the Rizzoli model [1,2] are shown in Table 3.

### 2.8. Statistical Analysis

Standard-related validity [23] of the joint angles for each foot model was examined using Pearson’s correlation analysis between the joint angles of each foot model and the true value. Determination of whether there was systematic bias of each foot model was examined using Bland–Altman analysis [24] between the joint angles of each foot model and the true value. Comparisons between the mean and peak joint angles of the new models and the Rizzoli model were made using unpaired t-tests, with a significance level of 5%.

## 3. Results

### 3.1. Experiment of Dummy Foot

**Figure 5:**
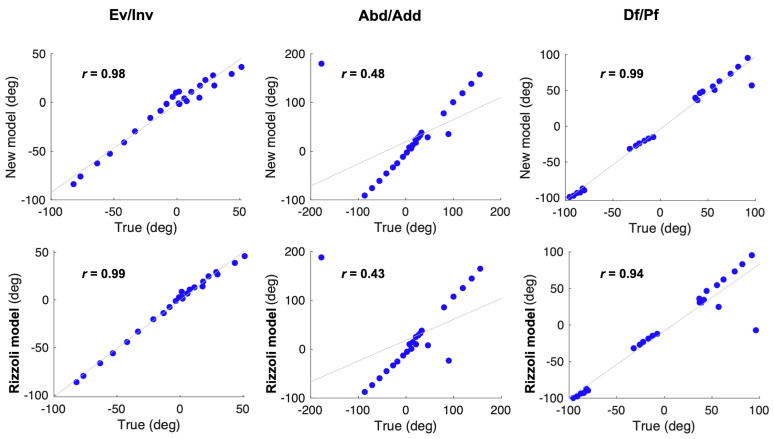
Univariate analysis using Pearson’s correlation analysis. Left to right, the Met_Hal angle in the frontal, transverse, and sagittal planes are shown. Correlations were assessed between the true values and the calculated values according to the two models; our new model (first row) and the Rizzoli model (second row).

**Figure 6:**
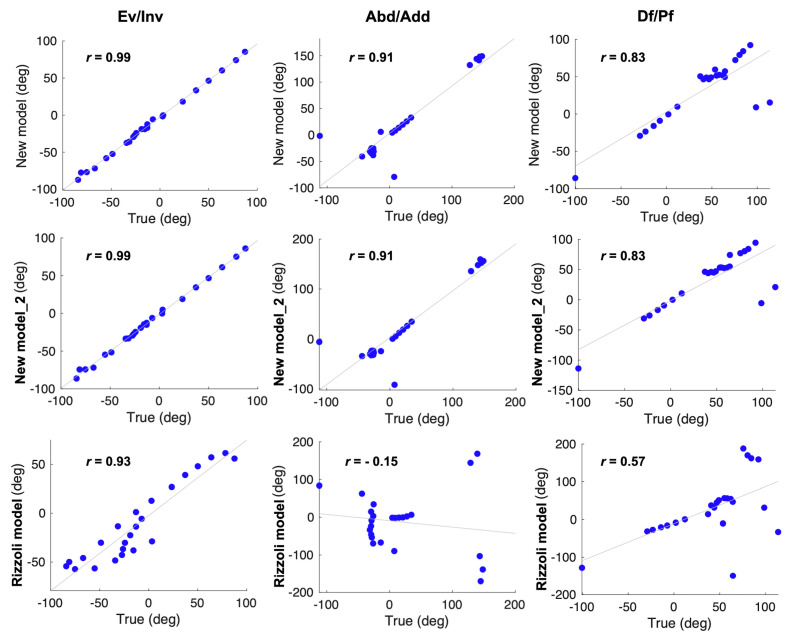
Univariate analysis using Pearson’s correlation analysis. Left to right, the Cal_Met angle in the frontal, transverse, and sagittal planes are shown. Correlations were assessed between the true values and the calculated values according to the three models, respectively; our new model (first row), our new model_2 (second row), and the Rizzoli model (third row).

**Figure 7:**
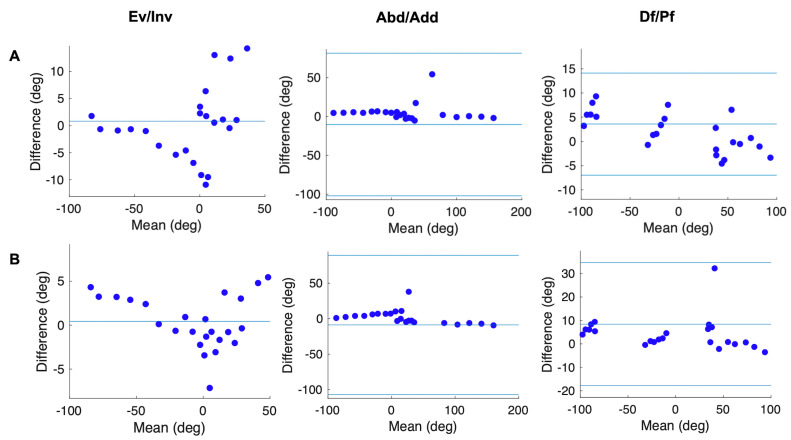
Bland-Altman plots showing the differences between the true values of Met_Hal angle and Met_Hal angle calculated according to the two models: (**A**) our new model and (**B**) the Rizzoli model, against their means. When they do not have either a fixed or proportional bias, the mean is shown (one blue solid line). When they have a fixed or proportional bias, the mean (the middle one of three blue solid lines) and limits of agreement are shown (the outer two of three blue solid lines).

**Figure 8:**
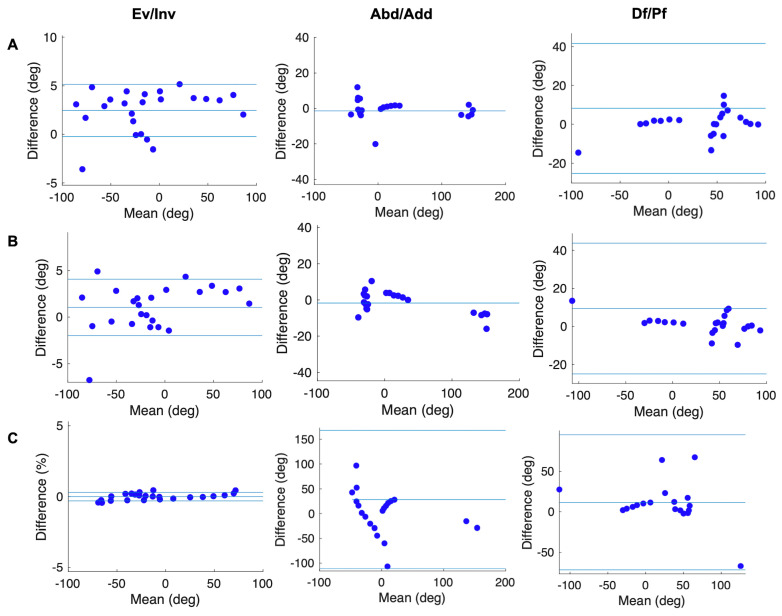
Bland–Altman plots showing the difference between the true values of the Cal_Met angle and the Cal_Met angle calculated according to the three models; (**A**) our new model, (**B**) our new model_2, and (**C**) the Rizzoli model, against their means. When they do not have either a fixed or proportional bias, the mean is shown (one blue solid line). When they have fixed or proportional biases, the mean (the middle one of three blue solid lines) and limits of agreement are shown (the outer two of three blue solid lines). When they have a proportional bias, the difference (Y-axis) calculates the relative value to the mean (X-axis).

Pearson’s correlation analysis (Figure 5 and Figure 6) showed that the joint angles calculated according to our new models were strongly positively correlated with the true values (*r* > 0.8, *p* < 0.01), excluding the Met_Hal angles in the transverse plane (*r* = 0.48). In addition, in the Bland–Altman analysis (Figure 7 and Figure 8), the Met_Hal angles in the frontal plane and the Cal_Met angles in the transverse plane did not have systematic bias. The other joint angles in the other planes had fixed biases; however, they were in the range of the limits of agreement, other than the Cal_Met angles in the frontal plane.

The joint angles were calculated according to the Rizzoli model, and the Pearson’s correlation analysis (Figure 5 and Figure 6) showed that the Met_Hal angles in the transverse plane and the Cal_Met angles in the sagittal plane were positively correlated with the true values (*r* = 0.43, *p* = 0.035, *r* = 0.57, *p* = 0.032, respectively). The Cal_Met angles in the transverse plane were weakly negatively correlated with the true values (*r* = −0.15, *p* = 0.48). The other joint angles in the other planes were strongly positively correlated with the true values (*r* > 0.9, *p* < 0.01). In addition, the Bland–Altman analysis showed that (Figure 7 and Figure 8) the Met_Hal angles in the frontal plane did not have systematic biases. On the other hand, the Cal_Met angles in the frontal plane had a proportional bias, and the other joint angles on the other planes had a fixed bias. These were in the range of the limits of agreement, except the Cal_Met angles in the frontal plane.

### 3.2. In Vivo Experiment

**Figure 9:**
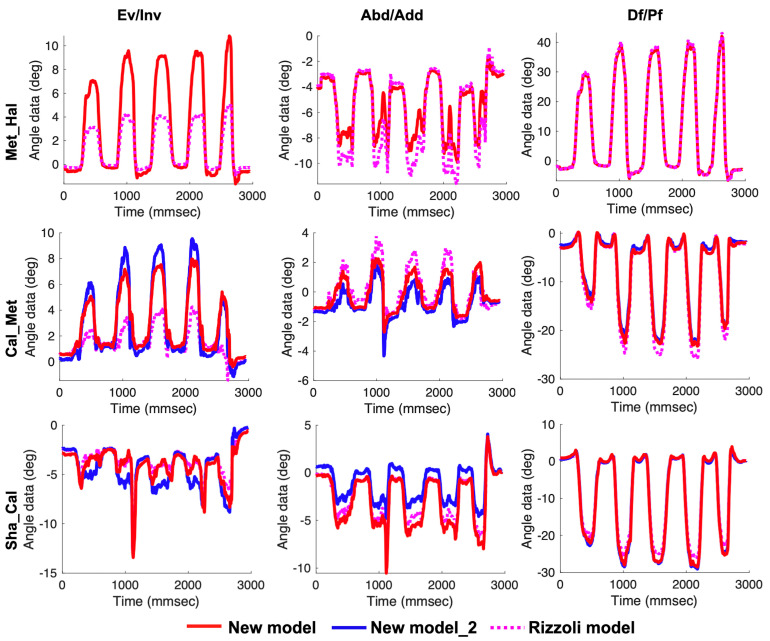
Left to right, the temporal patterns of rotation in the frontal, transverse, and sagittal planes during the calf raise. New model (red solid line), New model_2 (blue solid line), and the Rizzoli model (pink dot line) are shown. First to third row in order, the three joint angles (Met_Hal, Cal_Met, and Sha_Cal) are shown.

**Figure 10:**
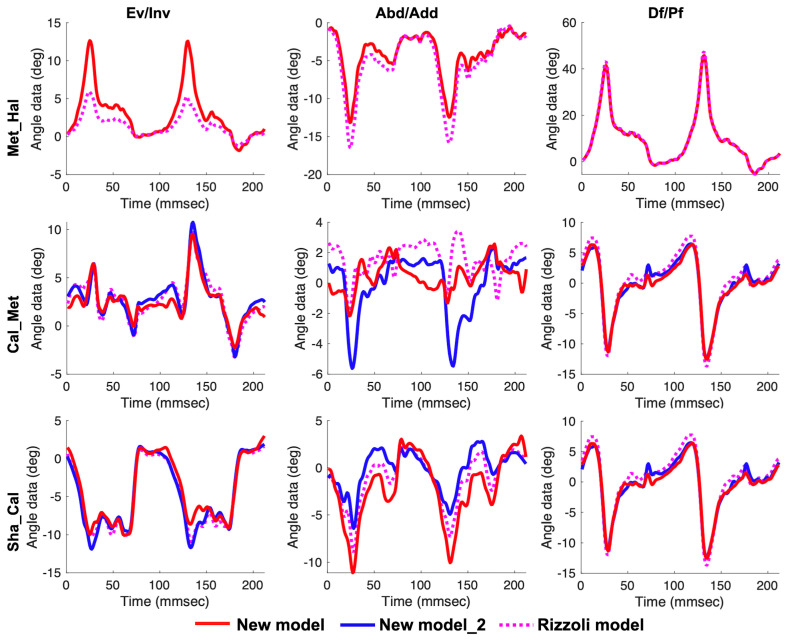
Left to right, the temporal patterns of rotation in the frontal, transverse, and sagittal planes during gait. New model (red solid line), New model_2 (blue solid line), and the Rizzoli model (pink dot line) are shown. First to third row in order, the three joint angles (Met_Hal, Cal_Met, and Sha_Cal) are shown.

**Figure 11:**
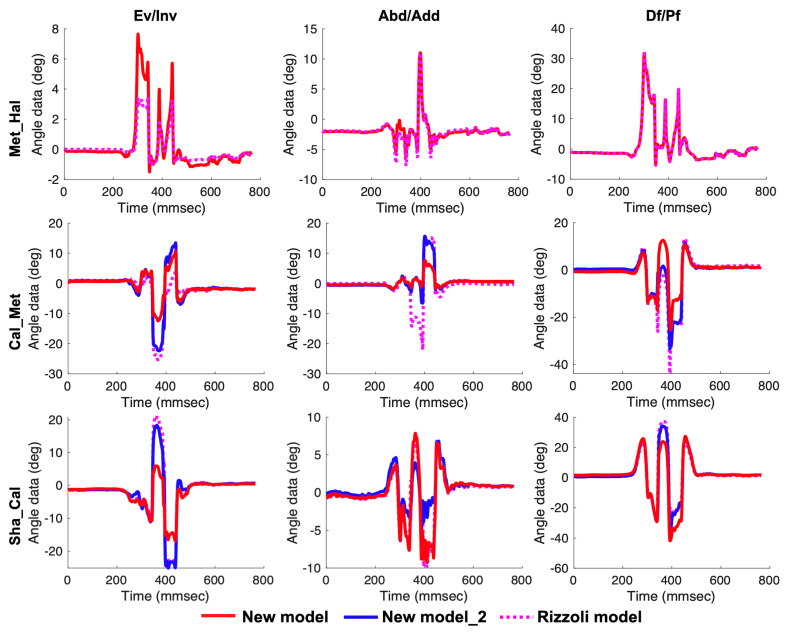
Left to right, the temporal patterns of rotation in the frontal, transverse, and sagittal planes during the drop jump. New model (red solid line), New model_2 (blue solid line), and the Rizzoli model (pink dot line) are shown. First to third row in order, the three joint angles (Met_Hal, Cal_Met, and Sha_Cal) are shown.

There were no differences in terms of joint angles in the static standing between our new models and the Rizzoli model. Temporal patterns during calf raise, gait, and drop jump are shown in Figure 9, Figure 10 and Figure 11, respectively. Temporal patterns of foot motion such as the Rizzoli model were observed in our new model during all dynamic tasks.

In Table 4 and Table 5, the peak joint angles of the dynamic tasks are displayed. During calf raise, the peak maximum joint angles of the Met_Hal angle in the frontal plane and the rearfoot relative to the shank (Sha_Cal) angle in the sagittal plane were significantly higher in our new model than in the Rizzoli model (*p* < 0.01). On the other hand, the peak maximum joint angles of the Sha_Cal angle in the frontal plane and the peak minimum joint angle of the Sha_Cal angle in the frontal and sagittal planes were significantly lower in our new model than those in the Rizzoli model (*p* < 0.01).

During the drop jump, the peak maximum joint angles of the Met_Hal angle in the frontal plane and the peak minimum joint angle of the Cal_Met angle and the Cal_Met angle_2 in the transverse and sagittal planes were significantly higher in our new model than in the Rizzoli model (*p* < 0.01). On the other hand, the peak maximum joint angles of the Cal_Met angle and the Cal_Met angle_2 in the sagittal plane and the Sha_Cal angle in the frontal plane were significantly lower in our new model than those in the Rizzoli model (*p* < 0.01).

On average, the peak values of our new model during dynamic tasks on the frontal, transverse, and sagittal planes were within the standard deviation of 2.7 degrees, 2.9 degrees, and 4.8 degrees, respectively. In contrast, the peak values of the Rizzoli model during the dynamic tasks on the frontal, transverse, and sagittal planes were within the standard deviation of 2.7 degrees, 3.5 degrees, and 5.2 degrees, respectively.

## 4. Discussion

### 4.1. Validity of Our New Multi-Segment Foot Model

The standard-related validities and systematic biases of our new multi-segment foot model were quantified by analyzing a dummy foot using a 3D motion capture system and by defining the true values by calculating the 3D rotation angles of the plates on which it was placed. Pearson’s analysis showed that most joint angles calculated according to our new models were strongly positively correlated with the true values. The Met_Hal angle on the transverse plane had a weaker positive correlation than the other 3D rotation angles; however, this could have been affected by the gimbal lock [25,26] because the joint angle was calculated for the Euler angle.

Systematic bias was examined using the Bland–Altman analysis. Our new models had a fixed bias in most 3D rotation angles. Values including fixed bias are required to perform a zero calibration. However, we could not perform a zero calibration because the measurement accuracy of the true value was not examined. Most of the 3D rotation angles including a fixed bias were within the range of the limits of agreement; therefore, applying our new models to a 3D motion capture did not present statistical difficulties. For a living body, the range of motion of the foot is narrower than that of the major joints. When performing the 3D motion analysis during movements, the range of motion of the foot ranged from −45 to 45 degrees, even for the joints that move relatively large amounts. We concluded that our new multi-segment foot model was valid for the motion analysis of direction or the amount of displacement within the range of motion during dynamic movements. In addition, two segmentation patterns of the rearfoot were examined and they were almost identical. We concluded that the rearfoot had the same validity, even for a difference of segmentation in our new foot model.

In this study, the validity was verified using Pearson’s correlation analysis and Bland-Altman analysis for the 3D rotation angles calculated by the Rizzoli model and the true value. We found that the correlation between the dorsiflexion angle of Cal_Met and the true value was lower than that for the new model, and the abduction angle of Cal_Met and the true value were negatively correlated. Regarding the Rizzoli model, many studies have reported reproducibility in gait analysis [1,2,10,27]. Leardini proposed the usefulness because it was highly reproducible among the subjects, and consistent with clinical and biomechanical knowledge [1]. We believe that the usefulness of the multi-segment foot model was confirmed by the fact that the truss mechanism [28] and windlass mechanism [29], which are the foot functions that cannot be observed with the single-segment foot model, were confirmed. However, few reports have verified the measurement validity by using a dummy foot as in this study. In the present study, the 3D rotation angle of each plate put on each dummy foot was treated as the true value, and the range of motion was considered from −90 degrees to 90 degrees; however, this range of motion does not occur in the living body, despite movement in all planes. Therefore, we presumed that the validity was low even with the generalized Rizzoli model. The statistical results of this study indicated that the Cal_Met angle in the transverse plane calculated according to the Rizzoli model needs to be treated carefully.

Our new model was more valid in the kinematic analysis than the Rizzoli model. However, skin artifacts could affect the measurement value as the limitation of 3D motion capture after being applied for the living body using our new foot model [18,30]. The skin artifacts in 3D motion capture have been reported at 31 mm in the thigh [31] and 23 mm in the shank [32]. They have also been reported in the toe off phase during walking; when the ankle joint undergoes maximum plantar flexion, the skin artifacts occurred at 16 mm in the midfoot and 12 mm in the hindfoot [18]. In addition, approximately 70% of the angles of the transverse tarsal joint and Lisfranc joint have been reported to show an error of approximately 5 degrees during the verification of the skin artifacts using the foot model [17]. Considering this effect or error, we need to perform more measurement trials and carefully treat measurement values when using our new multi-segment foot model.

### 4.2. Characteristics of Our New Multi-Segment Foot Model in Measuring Dynamic Tasks

Characteristics of our new multi-segment foot models during dynamic tasks were examined using a 3D motion capture by comparing the 3D rotation angles calculated according to our new models and the Rizzoli model. Unpaired t-tests revealed that the mean 3D rotation angles in static standing and temporal patterns showed no differences; however, the peak value of some 3D rotation angles were significantly different. Moreover, the peak value of our new model was within the standard deviation of 4.8 degrees, while that of the Rizzoli model was within the standard deviation of 5.2 degrees. Previous studies have reported that the joint angle of the Rizzoli model was within a standard deviation of 7 degrees in the gait analysis of healthy young participants [1,2]. It could be said that the results of this study were comparable to those of the previous studies. An angle of 5 degrees might have a significant effect on the joint in the foot with little motion. However, using the Rizzoli model has been reported to correspond to clinical and biomechanical knowledge not only for walking but also for landing [33] and running [34], suggesting that it contributes to the evaluation of foot function. Therefore, we found our new multi-segment foot model to be useful for analyzing foot motion using a 3D motion capture during dynamic tasks, because the temporal pattern was the same as the Rizzoli model that is generally used.

A previous study that examined the differences of multi-segment foot models reported significant differences in the kinematic patterns and peak values, and these differences depended on the location of tracking markers using the rigid-body assumption [22]. Our new model did not include the midfoot compared to the Rizzoli model; therefore, the range of motion of the forefoot relative to the rearfoot, both in our new model and in our new model _2, is presumed to have been attenuated.

Furthermore, in our new model, the range of motion of the rearfoot relative to the shank appears to have been amplified because the tracking marker of the rearfoot was located more distally than in the Rizzoli model. In contrast, in our new model_2, the range of motion of the rearfoot relative to the shank and showed no significant differences from the Rizzoli model as with our new model, because more tracking markers of the rearfoot were located proximally than in our new model. Differences observed with the multi-segment foot depended on the location of tracking markers, as in the previous study. We clarified the characteristics by analyzing the dynamic tasks of our new multi-segment foot model. We concluded that it was important to understand the characteristics of the multi-segment foot model during analysis, because the calculated values changed depending on the method of segmentation. The other multi-segment foot models were not evaluated for validity or reliability in as detailed a fashion as in this study. Moreover, our new model was validated against a general foot model even with dynamic tasks, and results were obtained that were useful for the study of the foot kinematics using motion capture. Compared with other foot models based on these points, we believe that our new multi-segment foot model has a higher degrees of usefulness and versatility and can be applied to examine both foot kinematics and kinetics.

### 4.3. Methodological Limitations

In this study, the validity of our new multi-segment foot model was investigated using a dummy foot. Measuring a living body, it is difficult to investigate the validity of the 3D rotation angles with a fine angle setting beyond the range of motion of the body. We treated the 3D rotation angles of each plate on which each dummy foot was placed as a true value, however, there is a limit of consistency because true value measurement accuracy was not examined in this study. Nevertheless, we believe that this study is novel because using the dummy foot can be objectively assessed, as the validity of the model itself, as well as the definition of the coordinate axes, calculation, and measurement accuracy. In the future, we will prove the usefulness of the model by evaluating the foot model when it is applied to the kinetics analysis.

In addition, it is a fact that inertial measurement units (IMU) [35,36] or wearable systems [37] have been developed as means of motion analysis. Motion analysis using reflection markers, as in this study, requires a large amount of expensive machinery and time, and also places heavy burdens on the subject; therefore, there is a limit in that it is more difficult to apply in daily life and sports movements than are IMUs or wearable systems. Nevertheless, the foot has less range of motion, and is affected by the impact from the ground and the attachment position of sensors [38]. Therefore, we believe that it is difficult to measure foot kinematics in detail using IMUs. In the future, while accumulating results demonstrating foot kinematics and kinetics using the motion analysis method, such as in this study, it may be possible to develop a simple measurement sensor such as an IMU or wearable system.

## 5. Conclusions

We defined a new multi-segment foot model that has three foot segments, to analyze foot kinetics and kinematics in the frontal plane in detail during dynamic movements. Using a dummy foot and the living body, we evaluated the validity, reliability and characteristics of our new multi-segment foot model and determined their differences according to segment definition. We confirmed that our new multi-segment foot model was valid for motion analysis for examining motion direction or the amount of displacement within the range of motion during dynamic movements. We found that our new multi-segment foot model was useful for analyzing the foot motion using 3D motion capture during dynamic tasks as well as the Rizzoli model, whether the rearfoot was defined as the transverse or sagittal plane. We concluded that our new multi-segment foot model has a high degree of usefulness and versatility, and could be applied to examine both foot kinematics and kinetics. Our new model might not only demonstrate foot motor function and disorder mechanism, but also might be able to contribute to design of braces and artificial limbs that assist foot function.

## Figures and Tables

**Table 1:** Marker location of our new model and marker name corresponding to the Rizzoli model.

Name	Description	For the Rizzoli Model
P1	Dorso-medial aspect of the first proximal phalanx head	PM
H1	Dorso-medial aspect of the first metatarsal head	FMH
H2	Dorso-medial aspect of the second metatarsal head	SMH
H5	Dorso-lateral aspect of the fifth metatarsal head	VMH
B1	Dorso-medial aspect of the first metatarsal base	FMB
B2	Dorso-medial aspect of the second metatarsal base	SMB
B5	Dorso-lateral aspect of the fifth metatarsal base	VMB
NV	Most medial apex of the navicular bone	TN
ST	Most medial apex of the sustentaculum tali	ST
PT	Lateral apex of the peroneal tubercle	PT
C1	Superior apex of calcaneus	CA (FCP)
C2	Apex of calcaneal tuberosity	HL (FCD)
LM	Distal apex of the lateral malleolus	LM
MM	Distal apex of the medial malleolus	MM
TT	Most anterior prominence of the tibial tuberosity	TT
HF	Most proximal apex of the head of the fibula	HF
MC	Midpoint between NV and B5	ID
IM	Midpoint between MM and LM	IM

**Table 2:** Segment reference frames defined by a primary axis, a plane.

Segment	Long Axis	Plane
Hallux	H1 to P1	H1, P1, H5 (Transverse)
Forefoot	MC to H2	MC, H1, H5 (Transverse)
Rearfoot	C1 to MC	C1, NV, B5 (Transverse)
Rearfoot_2	C2 to MC	C2, MC, C1 (Sagittal)
Shank	IM to TT	IM, LM, HF (Frontal)

**Table 3:** The definition of the abbreviations for the angle names.

Angle Name	Definition
Met_Hal	Hallux relative to forefoot
Cal_Met	Forefoot relative to rearfoot
Cal_Met_2	Forefoot relative to rearfoot_2
Sha_Cal	Rearfoot relative to shank
Sha_Cal_2	Rearfoot_2 relative to shank

**Table 4:** Peak angle calculated according to our new model. (Unit: deg.)

	Calf Raise/Maximum (SD ^1^), Minimum (SD)			Drop Jump/Maximum (SD), Minimum (SD)		
	Ev/Inv	Abd/Add	Df/Pf	Ev/Inv	Abd/Add	Df/Pf
Met_Hal	7.5 (1.9) *, −1.3 (0.8)	3.1 (3.1), −8.4 (4.2)	37.6 (7.7), −6.5 (4.0)	5.5 (1.8) *, −1.6 (0.6)	6.1 (2.5), −4.4 (2.6)	26.6 (4.9), −6.9 (3.9)
Cal_Met	6.2 (3.0), −2.3 (2.3)	3.6 (2.2), −3.0 (1.8)	2.5 (1.4), −16.8 (6.0)	4.1 (2.1), −7.1 (2.5)	2.8 (1.4), −3.7 (1.5) *	11.1 (1.9) *, −17.5 (5.1) *
Cal_Met_2	6.0 (3.0), −3.9 (3.1)	2.4 (1.8), −6.7 (6.3)	2.2 (1.3), −17.2 (5.4)	4.6 (2.8), −9.8 (4.6)	4.0 (2.5), −7.6 (2.3) *	12.4 (3.3) *, −17.9 (4.2) *
Sha_Cal	1.4 (1.8) *, −11.6 (5.2) *	2.0 (1.7), −14.6 (5.9)	6.1 (2.6) *, −35.9 (6.9) *	2.7 (2.3) *, −10.7 (3.3)	7.1 (2.4), −12.1 (3.7)	27.6 (5.4), −36.5 (5.8)
Sha_Cal_2	1.3 (1.3), −12.6 (5.4)	2.4 (1.3), −8.9 (4.4)	6.5 (3.1), −32.7 (6.5)	5.3 (3.6), −11.4 (3.5)	7.3 (2.3), −8.6 (3.8)	26.1 (5.2), −35.6 (7.0)

* Peak angles had a significantly difference compared to the Rizzoli model (*p* < 0.01). ^1^ Standard deviation.

**Table 5:** Peak angle calculated according to the Rizzoli model. (Unit: deg.)

	Calf Raise/Maximum (SD ^1^), Minimum (SD)			Drop Jump/Maximum (SD), Minimum (SD)		
	Ev/Inv	Abd/Add	Df/Pf	Ev/Inv	Abd/Add	Df/Pf
Met_Hal	3.7 (1.5), −1.1 (0.8)	3.6 (4.2), −9.3 (5.1)	38.4 (7.7), −6.6 (4.0)	2.8 (1.1), −1.4 (0.69)	6.4 (2.5), −5.2 (2.8)	27.1 (5.0), −6.9 (3.9)
Cal_Met	5.1 (3.8), −3.6 (3.6)	2.9 (2.5), −4.4 (2.7)	3.1 (1.7), −21.2 (7.1)	4.7 (2.5), −8.2 (4.1)	3.7 (3.1), −10.6 (5.2)	15.0 (4.1), −24.4 (6.9)
Sha_Cal	1.6 (2.2), −9.3 (4.1)	1.9 (1.5), −11.6 (5.3)	5.6 (2.4), −30.1 (6.2)	5.7 (4.1), −10.3 (3.4)	7.2 (2.4), −10.7 (4.1)	25.7 (5.3), −32.8 (7.9)

^1^ Standard deviation.
